# Device-Free Tracking through Self-Attention Mechanism and Unscented Kalman Filter with Commodity Wi-Fi

**DOI:** 10.3390/s23125527

**Published:** 2023-06-13

**Authors:** Kabo Poloko Nkabiti, Yueyun Chen

**Affiliations:** 1 School of Computer and Communication Engineering, University of Science and Technology Beijing, Beijing 100083, China; b20170607@xs.ustb.edu.cn; 2 School of Computing and Information Systems, Botswana Accountancy College, Private Bag, Gaborone 00319, Botswana

**Keywords:** target tacking, Channel State Information (CSI), Kalman filter, self-attention mechanism

## Abstract

Recent advancements in target tracking using Wi-Fi signals and channel state information (CSI) have significantly improved the accuracy and efficiency of tracking mobile targets. However, there remains a gap in developing a comprehensive approach that combines CSI, an unscented Kalman filter (UKF), and a sole self-attention mechanism to accurately estimate the position, velocity, and acceleration of targets in real-time. Furthermore, optimizing the computational efficiency of such approaches is necessary for their applicability in resource-constrained environments. To bridge this gap, this research study proposes a novel approach that addresses these challenges. The approach leverages CSI data collected from commodity Wi-Fi devices and incorporates a combination of the UKF and a sole self-attention mechanism. By fusing these elements, the proposed model provides instantaneous and precise estimates of the target’s position while considering factors such as acceleration and network information. The effectiveness of the proposed approach is demonstrated through extensive experiments conducted in a controlled test bed environment. The results exhibit a remarkable tracking accuracy level of 97%, affirming the model’s ability to successfully track mobile targets. The achieved accuracy showcases the potential of the proposed approach for applications in human-computer interactions, surveillance, and security.

## 1. Introduction

Recently, significant advances in embedded systems and radio have led to the emergence of ubiquitous device-free wireless systems, which have become an important research area. Since wireless networks are ubiquitous everywhere in the range of the transmitted signal, we would be interacting with radiofrequency (RF) electromagnetic (EM) waves [1,2,3,4]. The device-free-based wireless systems are used in different applications such as smart homes, monitoring industrial automation, medical applications, monitoring older adults in nursing homes, monitoring prisoners in custody, and target tracking [5,6,7,8].

Target tracking is an existing research area, and the application of device-free target location comprises the automatic position estimation of a moving target. We consider the target tracking problem to be a sequential localization problem. A real-time location is needed using a specific algorithm to estimate the moving target’s exact location. Usually, the radio transmitter (TX) broadcasts a signal within a network, and the target reflects the propagating signal [9,10,11]. When the signal is received by the receiver (RX), it can be used to estimate a target’s location [12,13,14,15]. Different techniques can be used for target tracking, which include the angle of arrival (AOA), received signal strength indicators (RSSI), the time-of-arrival (TOA), as well as the time difference of arrival (TDOA) [16,17]. Past research has pointed out that the TOA and TDOA can better estimate position than other methods [18,19]. The main drawback of these techniques is that they need costly hardware, making them impractical to apply in different applications [20,21]. On the other hand, we intend to adopt a method in our research study that only uses commodity Wi-Fi devices, a TP-LINK 450 access point as a transmitter (TX), and a gigabyte laptop receiver (RX).

Moreover, traditional approaches require subjects to wear sensors or transceiver-equipped, which are then localized to the sensors rather than the wearer through observing changes and the fluctuating received signals into a coordinate system [22,23,24]. The other drawback of using traditional methods such as received signal strength indicator (RSSI) compared to channel state information (CSI) is that RSSI measurements are per a single packet value [8,25,26,27,28]. In contrast, the CSI is more robust and stable and contains more data. Furthermore, CSI measurement is per every packet’s orthogonal frequency-division multiplexing (OFDM). Hence, the CSI applies to complex environments. Moreover, compared to the traditional RSSI, which only uses one measure of power in the entire channel bandwidth, the CSI presents the phase information along with amplitude within the multiple OFDM subcarriers [1,18,29,30]. The CSI measure from the physical layer is usually tracked from within through the IEEE 802.11 multiple-input multiple-output (MIMO), which comes with the recent commodity Wi-Fi devices. The collected CSI is fine-grained; thus, it can detect very slight movements at a millimeter level to recognize tiny movements. The CSI data acquired through commercial Wi-Fi devices are usually extreme in phase noises. Therefore, there is a need to impose restrictions on the scalability and then degrade the precision of tracking by examining the Doppler shift with the time–frequency analysis of the CSI [1,31,32]. The hardware imperfection usually causes this extreme phase noise within the CSI data [33]. Therefore, refining the dynamic phase, coming from an extremely noisy CSI, is challenging. Hence many studies have applied different machine-learning algorithms to solve this problem with varying success rates [18]. Most target-tracking algorithms employ the Gaussian process regression, which operates through position estimation. They would reconstruct a constantly uniform trajectory after that, and they would regain the missing positions [1,2,18].

Furthermore, these tracking algorithms also used the state-space model to improve the estimated position using information from the previous position. Nevertheless, the main drawback of these models is that they only work more effectively if the target they are tracking has a slight difference in velocity or acceleration. Other studies discussed the methods that are based on RSSI, which used some nodes for localization within device-free wireless networks. These methods focused mainly on getting the estimated location of a target by observing and investigating its information without using mobile notes. Many studies that adopted the RSSI-based model used the radio fingerprinting approach for tracking targets. This method permits the usage of static properties within an environment. Some studies on RSSI-based radio-fingerprinting adopted the Extended Kalman filter and the second-order state-space algorithm to track targets that performed exceptionally well [34,35].

This paper proposes a device-free target tracking method in Wi-Fi that combines channel state information and a self-attention mechanism. To the best of our knowledge, there has not been any study that solely uses the self-attention mechanism and CSI for indoor target tracking. The proposed method is created to react to hyperactive targets, which will help in increasing its accuracy for tracking a target. The proposed method also took advantage of the method that uses the target’s mobility to improve obtaining the estimated position of the target. Furthermore, the proposed system uses commercial Wi-Fi devices to track a moving target directly through the target’s instantaneous acceleration and CSI simultaneously, and then apply the sole-self-attention mechanism to compute the target’s position. The main critical contributions of this research study include the following:

- We propose a state-of-the-art fine-grained target tracking model that uses a sole self- attention mechanism and CSI to locate and track moving targets passively and estimate the position with the help of their instantaneous acceleration. Furthermore, the model uses an approach of processing through batches to estimate the target’s path;
- For tracking targets online, we employed particle filtering, improving the position estimation accuracy as time passed;
- We propose using the unscented Kalman filter to estimate the instant positions of moving targets;
- To validate the performance of the proposed model, we carried out some experiments in real-time by using commodity Wi-Fi devices on a custom-designed platform.

The structure of the entire paper is as follows: Section 2 is the Background and Related Work, Section 3 is the System Model, Section 4 is the Optimization based on the Position Algorithm, Section 5 is the Experimental Setup, Section 6 is the Simulations and Results, Section 7 Results Discussion and Section 8 Conclusion and Future Work.

## 2. Background and Related Work

Device-free tracking systems, vital for numerous applications ranging from indoor navigation to smart environments, leverage a variety of technologies, each providing unique advantages and addressing specific challenges. These technologies encompass Wi-Fi, RFID-based systems, sensor-fusion, GPS, and Bluetooth, among others. A novel approach to Bluetooth Low Energy (BLE) scalability is proposed by Tekler et al. aiming to overcome the challenges associated with its widespread implementation [36]. This study significantly contributes to the body of research in BLE technology, offering potential solutions for future high-density, low-power communication networks. While BLE excels in power efficiency and fits well within short-range connectivity applications, it lags Wi-Fi in aspects such as range, data rates, network complexity, and advanced security protocols. In the realm of RFID-based indoor location sensing, Li et al. present a comprehensive evaluation showcasing its efficiency and accuracy. However, it is pertinent to note that RFID technology, while effective, does not exhibit the robustness and versatility inherent in Wi-Fi systems [37]. Limitations such as shorter range, slower data transmission speeds, lack of seamless integration with internet services, and challenges in supporting high data throughput applications mark some of the significant disparities between RFID and Wi-Fi. Shifting the focus to predictive modeling, Low et al.’s study explore commercial vehicle activity prediction using a hybrid sampling and gradient-boosting approach to address imbalanced class distribution [38]. While this approach presents promising results, it underscores the inflexibility stemming from reliance on dedicated infrastructure, a stark contrast to the adaptability of Wi-Fi-based systems. Additionally, real-time data processing and the lack of versatility associated with internet connectivity could pose significant challenges. Tekler et al. delve into occupancy prediction across multiple space types using deep learning [39]. They propose a minimum sensing strategy that, while efficient, might suffer from lower data resolution compared to data obtained through comprehensive Wi-Fi sensing. Furthermore, the computational demands of deep learning may impact real-time prediction capabilities, and potential latency issues might arise when transmitting data over non-Wi-Fi networks. In conclusion, each of these technologies offers unique capabilities in device-free tracking applications. However, when compared to the versatility and robustness of Wi-Fi, each presents distinct limitations that need to be addressed to realize their full potential.

Regarding device-free tracking with commodity Wi-Fi, different machine-learning techniques have been used in various studies [1,2,40]. This section reviews different approaches for tracking targets indoors using a wide range of applications with commodity Wi-Fi. Research on Multi-target tracking using the Probability Hypothesis Density (PHD) filter with radar was conducted by Chen et al. They asserted that their proposed algorithm obtained high tracking accuracy while also achieving high computational efficiency [41]. Zhichao et al. proposed a state-of-the-art algorithm for tracking weak maneuvering targets using the Bayesian theory with multiple models (MM) based filter. They claimed that the simulation results indicated that the proposed model obtained outstanding results and was more effective than other traditional models [42]. Furthermore, Gongguo et al. proposed a new method for scheduling sensors for the targets that maneuver on the ground and track them in a blind spot zone. The model utilized the Markov decision process (POMDP) framework, and the results demonstrated that the proposed method has a better target tracking accuracy than traditional methods [43,44]. Wang et al. used the Doppler shift from the Wi-Fi signal to extract features they used to perform the action recognition [45]. They further used a deep learning model to better the performance of the proposed system. Their adopted methods had to learn their highly sensitive environment’s properties through wireless signals [33]. Wang et al. further explored transfer learning algorithms using Wi-Fi signals to train different feature modeling on different scenarios. All the proposed systems performed significantly well. However, the feature extraction processes for these systems were highly computational and time-consuming, and they required a lot of resources to run [46]. Mahfouz et al. used deep learning with a Kalman filter for targets in wireless sensor networks [2]. They compared two machine learning models, the ridge regression and the vector output regularized least squares. Their findings indicated that the prediction of the position of the targets while using its acceleration information and the estimates that come first leads to accurate tracking. They also claimed that their proposed model demonstrated more robustness when dealing with noisy data than traditional tracking methods. Shi et al. proposed a novel idea of using device-free channel state information data to track moving targets using Probabilistic Fingerprinting [1] accurately. Their findings also indicated that the proposed Cramer–Rao Bound system had a lower mean square error. They compared the Kalman filter and Bayesian filter estimators, and the Kalman filter estimator proved to be a more optimal estimator when used in linear systems.

Bybordi and Reggiani proposed using the extended Kalman filter with fingerprinting for indoor tracking targets. Their results indicated that the proposed hybrid target tracking algorithm significantly improved the system’s performance. Nevertheless, the different environments affect the fingerprints, which their proposed algorithm neglected [47,48]. Wang et al. proposed an Optimal Target Tracking system based on Dynamic Fingerprint (OTTDF), and they employed the modified PF algorithm and the dynamic fingerprint update mechanism. They concluded that the proposed algorithm had two advantages over traditional tracking methods: The first was refining the sampling method by thus reducing the frequency of the sampling. The second reason was the dynamic updating of the fingerprints [5,12,49]. Qian et al. [20] proposed using CSI to detect moving targets, and their results indicated that in comparison to the previous approaches, their proposed approach is more robust and more accurate. Sun et al. [50] developed a deep learning model that estimated the parameter of multiple targets’ motion parameters used to perform detection and association in an end-to-end manner. For this research study, they used videos to track moving objects. Luo et al. [16] proposed a cooperative target tracking and positioning model which employs wireless sensor networks for indoor environments. Their model had three methods the overlapping mechanism, the prediction mechanism, and the cross-grid strategy. They concluded that the proposed system achieved higher accuracy and performance compared to a single network-based system. Zhang et al. [19] proposed a novel system that can locate the target device’s position freely. Most device-free tracking systems rely on machine learning algorithms, making them more labor-intensive. The proposed model simultaneously estimated the velocity of the multiple moving targets using angle-of-arrival and tracked targets’ close decimeters. They concluded that the proposed system DFT-JVAE achieved a good performance. Savazzi et al. [51] proposed a device-free method for tracking moving people using wireless networks and a stochastic log-normal RSS model. They used the fading effects of mobile targets for the indoor environment. Their findings indicated that the proposed system uses existing Wi-Fi commodity devices and standards more cost-effectively. They also concluded that the proposed system with PF Bayesian provided much better accuracy than traditional methods, reducing the sensitivity. Wilson et al. [52] proposed a device indoor localization schema using a statistical model that estimates mobile and static targets using Wi-Fi received signal strength (RSS) measurements. They argued that existing systems could not locate targets in environments with heavy obstruction. Their proposed fade-level skew-Laplace model and particle filter indicated that tracking targets become more accurate when a line of sight (LOS) exists than when no LOS exists.

Li et al. [22] proposed a target-tracking algorithm for outdoor targets that use an arrival angle to estimate the moving target’s position. They adopted the received non-coherent signal strength differences (RSSDs) model. The experimental results indicated that the proposed model could accurately predict the tracking of targets when there was no line of sight, and it had a very low computational complexity. Han et al. [34] proposed an accurate localization and target-tracking model using the KNN classification algorithm with Wi-Fi fingerprinting in indoor environments. They compared it with traditional machine learning models such as SVM, logistic regression, and random forest. The results indicated that the proposed systems significantly improved tracking and positioning accuracy. Shi et al. [1] proposed a device-free localization and target-tracking system that uses channel state information and probabilistic fingerprinting. The experimental results of the proposed system indicated that it could precisely predict the position of a moving target even when there are a lot of multipath effects within clustered indoor situations. They also used Bayesian filtering to track a moving target’s trajectory accurately. Bao et al. [5,53] proposed a Shadow-Enhanced Self-Attention and Anchor-Adaptive Network for Video SAR Moving Target Tracking. They proposed tracking targets using its shadows because it does not have a location shift, and the backscattering characteristic is stable. They claimed that the results acquired by their model achieved 83% accuracy. Even though this study adopted the self-attention model for target tracking, their main drawback was still some interruptions in their trajectories and reliance on video.

### Tracking Approach

**Figure 1:**
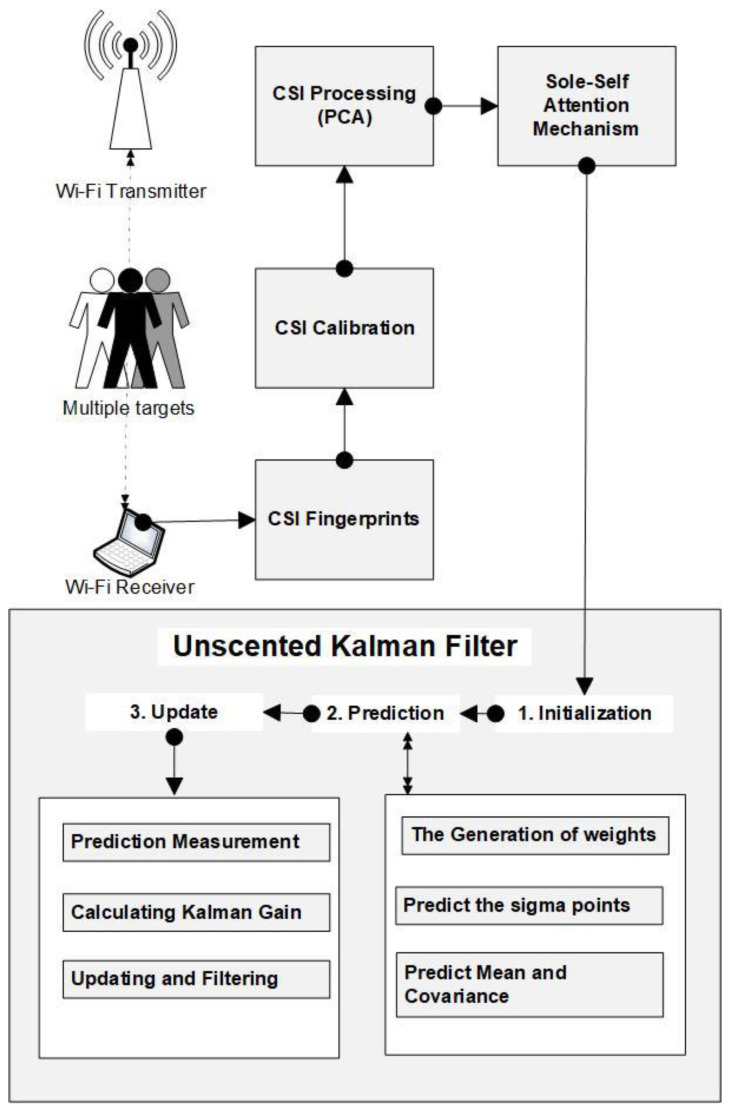
The Device-free target tracking with Sole Self Attention Mechanism and Unscented Kalman Filter.

The proposed method allows tracking several moving targets, and each target is tracked independently using their acceleration and the CSI data from commodity Wi-Fi devices. The proposed model is in a linear-state form that describes the target’s motion. Taking into consideration a 2D dimensional environment where the stationary access points $a_{p}$ and the target’s current $T_{l}$ locations are known and represented by $a_{p},p\in \left\{1,\ldots ..,T_{l}\right\}$. The target coordinates are presented in a D-dimension format and represented as vectors in a row. To adhere to generalization, a single target with known position pos(tm) is observed, whereby tm represents the current time step. The expression below depicts the motion of a target in the proposed model.

(1) $$pos\left(tm\right)=pos\left(tm-1\right)K+C\left(tm\right)+\theta \left(tm\right)$$

whereby the former position of the target is represented by $pos\left(tm-1\right)$, the transition matrix states are shown by K, which is D-dimensional and represents the target’s current position; on the other hand, $C\left(tm\right)$ is used to indicate the vector, which is utilized as an input control based on how the target accelerated. The $\theta \left(tm\right)$ is vectors noise and is considered to have a normal probabilistic distribution with a 0 mean. The R(tm) is a convenience matrix which means θ(tm)∼N(0.R(tm)). In addition to acceleration, other information is collected by the commodity Wi-Fi transmitter and receiver concerning the target within the network, such as the time step for every movement. The process of the entire target tracking with Wi-Fi CSI is defined in more detail in Section 3, where different system model processes are explained step by step, as indicated in Figure 1.

## 3. System Model

The architecture of the proposed system is illustrated in Figure 1 above. Firstly, the CSI fingerprint data is collected from the commodity device’s Wi-Fi network, the transmitter, and the receiver. The raw CSI data will go through the calibration process and then into the principal component analysis (PCA) method to decrease the dimensionality of large data. The proceeds will then be fed into the sole self-attention mechanism model, which estimates the target’s present location with regression. After that, the proposed deep learning model will pass its results to the unscented Kalman filter, updating the location using the previous location and the target’s current location. For accurate trajectories of the destination, mapping and pairings are applied to the filter. A detailed description of the model is discussed below.

### 3.1. Data Collection

The availing of the CSI tool by Halperin enabled us to capture the CSI from commodity Wi-Fi devices. This tool aggregates the phase information data and the amplitude for all the subcarriers, assuming that tr represents the number of transmitters TX. At the same time, the receiver re is RX antennas, while the total number of subcarriers is represented by y for both the TX and RX through the CSI tool. We were able to obtain the CSI vectors per packet that contained the values of tr⋅re⋅y from the sub-channels represented as $C=\left\{C_{a,ke}^{mt}\right\},a\in [1,tr],ke\in [1,re],m\in [1,y]$. Each value $C_{i,ke}^{mt}$ is a true reflection of the phase information and the amplitude of the subchannel from the propagating radio frequency signals $C_{a,ke}^{mt}=\left|C_{a,ke}^{mt}\right|e^{j\sin\theta }$, which are altered on the sub-channel mt being transmitted from the antenna a and then received by antenna ke. The CSI data was collected in two different indoor places, the School of Computing boardroom and the Hallway at the University of Science and Technology Beijing. Three participants were used as multiple targets. There are a lot of courses of errors and biases that might come from collected data, such as noise. For instance, the phase information data from the CSI information might contain many random noises compared to amplitude data [32]. The CSI data received by all the antennas at time te was defined as follows;

(2) $$\begin{array}{ll} R_{i}(te) & =U(\theta )L(te)+Ns(te),i=1,2,3\ldots Me\\ & =\left[v_{1}\left(\theta_{1}\right),v\left(\theta_{2}\right),\ldots ,v_{Ns}\left(\theta_{Ns}\right)\right]Si(te)+Ns(te) \end{array}$$

whereby U(θ) is a representation of tr dimensionality matrix, and L(te) denotes the signal arrival matrix, which can be further expressed as $Sg\left(te\right)={\left[s_{g1}(te),s_{g2}(te),s_{g3}(te),\ldots ,{s_{g}}_{Ns}(te)\right]}^{T}$, then the representation of the measurements is denoted as $R_{i}\left(te\right)={\left[R_{i1}(te),{R_{i}}_{2}(te),{R_{i}}_{3}(te),\ldots ,s_{g}tr(te)\right]}^{T}$. Then, there is Ns(te) which denotes a vector of the Gaussian noise. Lastly, there is $v_{i}\left(\theta_{i}\right)$, which the controlling vector represented as

(3) CSIi′=csi1,T′icsim,T′,…,csiNt×Nr,Tiθi=1e−j2πspsin⁡θi/λe−j2π(2sp)sin⁡θi/λ…e−j2π(Me−1)spsin⁡θi/λ

The speed of the propagating signals is about the same as the speed of light ls, and the spacing of antennas is defined as sp=λ/2, whereby the wavelength is represented as λ. After collecting the raw CSI data, we calibrated it before feeding it to the proposed model. We used both phase information and amplitude data in our proposed system.

### 3.2. CSI Processing (PCA) 

After collecting the raw CSI data, we pre-processed it to prepare it to be fed in the sole-self-attention mechanism model. The usage of the commodity network interface card along with the modified device drives to collect through grouping the channel frequency response into 30 subcarriers of $N_{t}$ in the layers that are at the top and could be formatted as follows for each packet:

(4) $$H=\left[H\left(f_{r1}\right),H\left(f_{r2}\right),\cdots \cdots ,H\left(f_{Nt}\right)\right]$$

Both the amplitude and phase information of the CSI from the OFDM subcarrier are denoted as follows:

(5) $$H\left(f_{r}\right)=∥H\left(f_{r}\right)∥u^{o∠H\left(f_{r}\right)}$$

whereby the representation of the CSI is $H\left(f_{r}\right)$, which is from a particular subcarrier and is denoted by r(r∈[1−30]), and the frequency is set at $f_{r}$, where the phase is represented by $∠H\left(f_{r}\right)$. For clarity, the phase is also represented as $\phi_{r}$. For the observation of the testing area, we employed a mechanism that collected the CSI consecutively us r as our measurement in a specified window period, and a sequence of CSI data is formed as indicated in the equation below;

(6) $$H=\left[H_{1},H_{2},H_{3},H_{4}\cdots ,H_{r}\right]$$

The algorithm we used for detecting the movement employed the r measurements from the CFR, which were then used as the input for the algorithm. The data processing process helped reduce the sparsity of the data, which helped in the learning process of the proposed model. We devised a strategic plan to obtain more stable CSI data by reducing the indoor CSI signal oscillation amplitude or attenuation. We adopted the principal component analysis (PCA) for noise cancellation and data dimension reduction since it has been proven to be more effective than other noise removal techniques for CSI signals, such as Butterworth filtering. The main reason for adopting PCA was to reduce complexity during implementation [46]. For full detail of the proposed model, we briefly described a critical process for the data pre-processing and calibrating the phase information [54,55,56].

### 3.3. CSI Calibration

This second sub-section highlights how the raw CSI data was calibrated. The altered device drivers used three antennas from the network interface card (NIC), which employed 30 sub-carriers out of the present 56. The main reason for calibrating the collected raw CSI data, both phase and amplitude, is the commodity hardware imperfections. Random phase offsets introduce errors in data such as sampling frequency offset (SFO), packet detection, and delay (PDD). Since the hardware lacks requisite qualities, it causes problems and errors in the phase information data. The reason for calibrating the raw phase CSI data is to overcome the sampling and carrier frequency offsets. The sampling frequency offset is a product of analog, which creates some unsynchronized clocks. This problem is challenging because offset phase error from the sampling frequency is specific and unique for every sub-carrier. Contrary to the carrier frequency offset, which is mainly caused by the signal down converter that is used to receive the radio signals, the main reason it happens is because of the inaccurate synchronization in the central frequency between the transmitter and the receiver. We cannot use raw CSI data directly for tracking targets. Hence, there is a need to calibrate it first before being processed. We presented the measured phase information for a particular sub-carrier as ∠Sud^i, which indicated the phase data collected from sub-carrier i. The expression can be shown as follows;

(7) ∠Sud^i=Sud^i+2πnmiFqΔtl+β+Ns

whereby denotes ∠Sud^i denotes the phase information. Because sampling frequency offset (SFO) makes the time or clock idle, we used the delta and tl to represent time (clock) Δtl. Then, there is a $nm_{i}$ that denotes the index of the sub-carrier for the sub-carrier number i. Moreover, there is Fq, which represents the fast Fourier transform (FFT). The beta β was also used to represent the offsets, which is unknown because of the carrier frequency offset (CFO) [57]. The noise measure was represented by Ns. The indices of $nm_{i}$ that may be from 1 to 30, along with the FFT, which is of size Fq, were attained from the 802.11 n standard specifications. Since Δtl and β are unknown, it hinders obtaining the actual phase data. However, this drawback was solved by using a linear transformation model on the raw phase CSI data obtained from the frequency bands, thereby doing away with Δcl and β with the assumption that ***f*** is the phase slope, and the t is the frequency band s entire offset. We observed that the $2\pi \frac{w_{i}}{Nq}\Delta t+\beta$ phase error is a specific linear function from the sub-carriers index.

### 3.4. The Fingerprint Generation

For this subsection, we briefly describe generation fingerprint radio maps using the calibrated phase information of the target that has been collected, which tracks moving targets in an indoor area [12,58]. Several fingerprint mapping methods have been proposed, as indicated by the literature. The fingerprints map was generated from the calibrated phase data for tracking targets during online and offline training. We adopted the value-based and site-survey fingerprint maps technique because it is extensively used in tracking systems and has been more stable. Although site surveying is very reliable when capturing data for stationary subjects, it is more likely to be choked by higher demand for manpower and time-consuming. Since both the amplitude and phase information varies significantly while a target is in different locations, we exploited this to perform the tracking of a target. We used the CSI feature maps to obtain a specific location of a moving target by matching the fingerprint to a matching location and plotting it as a feature map. From the rows of the $N_{q}$, we choose 100 at random of the 2D (dimensional) matrix numbers that are imaginary, being $csi_{c,N_{q}}^{s}$ to create 2D metrics of $100\times N_{t}$, for the restoration of the information about the position, which is set at reference point i−th of $CSI_{s}^{\prime }$. The expression for the scenario above is indicated below;

(8) $$CSI_{s}^{\prime }=\left\{csi_{1,L}^{\prime s}s_{1},\ldots ,csi_{c,L}^{s},\ldots ,{csi}_{N_{w}\times N_{e},L}^{s}\right\}$$

whereby the matrices of $100\times N_{t}$, is the imagined number represented by $csi_{c,L}^{s}$ for the c−th link of the i−th reference point ${RF}_{i}$. We used both actual and imaginary matrices numbers within the position information set $CSI_{s}^{\prime }$ to obtain the exact feature map, which creates the amplitude and phase data used to create feature maps set at $\Phi_{i}$ within $N_{w}\times N_{e}$ linked with ${RF}_{i}$. Moreover, the feature maps were obtained through the amplitude and phase data set at Φ within the reference points MP.

(9) $$\Phi_{i}=\left\{\phi_{1,t}^{i}\cup \cdots \cdots \cdots \cup \phi_{y,t}^{i}\cup \cdots \cup \phi_{N_{w}\times N_{e},t}^{i}\right\}$$

whereby the feature maps of the amplitude t were represented by $\phi_{y,t}^{i}$ with the y−th link at the reference point number i−th, wherein the $N_{w}\times N_{e}$ links the feature maps of the t amplitude, and phase was represented by $\Phi_{i}$ within the reference point number i−th.

### 3.5. Sole-Self Attention Mechanism

When the data and the fingerprint database are ready after the pre-processing process, it needs to be fed into the sole-self-attention mechanism machine learning model, combined with the unscented Kalman filter to help estimate the instant positions of a moving target. We used the collected CSI fingerprint database to define our proposed model, and the input was the phase information and the amplitude data whose output corresponds to the exact position. To evaluate our model, we compared it with another learning model, the ridge regression model. Then a moving target will measure the phase, amplitude, and acceleration. We first obtain the estimated current position of a target by using the self-attention mechanism model and the measured phase and amplitude. We then consolidate them with the target acceleration information with the help of the Unscented Kalman filter for better accuracy. So far, we have reviewed two different tracking models. The hyper-active target seems much more easily tracked with the proposed system than the less-active ones. The proposed sole self-attention mechanism is based on the Transformer model, a neural network architecture initially designed for natural language processing (NLP) tasks such as language translation and text generation [59]. We modified the sole self-attention mechanism to accommodate time series data inference tasks by encoding its key-value pairs and computing attention weights to predict future values without explicit time-series modeling techniques. Using a self-attention mechanism solely without recurrence nor convolution has performed well on language tasks, but few studies have explored it for object-tracking prediction tasks. The main reason for adopting the sole self-attention mechanism for tracking mobile targets was its ability to capture long-term dependencies and patterns in the data more efficiently while being computationally efficient and parallelizable, making it suitable for large-scale time series prediction of the future trajectory of moving target tasks. The literature shows that most studies utilized sequence-to-sequence models, such as Long Short-Term Memory (LSTM), to predict the future locations of targets by employing multi-step forecasting techniques. Since our proposed model used numerical data as input, there was no need to apply the embedding because it is usually used for natural language processing tasks. Hence, we transformed the 11-dimensional data into an n-dimensional space using a linear transform process. We adopted auto-regression for our tasks as it is not a word classification. The prediction of the current sequence needs information about the past arrangement. Our proposed system is a supervised learning architecture that employs SoftMax for learning the embedding used for converting the input tokens and the output tokens to dimensional vectors $d_{\;\mathrm{SAMEK}}$. Then the results were passed through the SoftMax method. The vectors’ value is then computed as the obtained score in the equation below.

(10) $$Attention\left(Q,K,V\right)=\mathrm{softmax}\left(\frac{QK^{T}}{\sqrt{d_{k}}}\right)V.$$

We then summarized the vectors of the weighted value that we applied as the output for the attention layer of our model at a distinctive position. Furthermore, we added a distinguished multi-headed attention mechanism into the preceding self-attention layer. The number of stacks of self-attention layers for our model was four layers. The model employed overlapping windows of a fixed length of 20. The foremost reason for disposing of this mechanism was to assist the proposed model in concentrating on unconventional positions. Various sets of weights matrices initialized randomly Q/K/V could be accessed through the help of multi-headed attention. As for the SoftMax function, we applied it with a learning linear transformation model employed for conventional decoding of the output, thus predicting the probability of a token that follows, depicting the current real-time position of a target being the reference point. We then multiplied the embedding layers inside the weights with by $\sqrt{d_{\;\mathrm{SAMEKI}\;}}$. During the weight training phase of the proposed model, the collected CSI data phase and amplitude were used as reference points for the offline and online phases. When training, we used two procedures which were fine-tuning and pre-training. The pre-training of the proposed model used data that was not labeled for different pre-training tasks. The fine-tuning of the proposed model was performed first by pre-training the parameters. We fine-tuned every parameter using labeled data from the tasks performed in the stream. For the deep bidirectional process training, a specific percentage of the input tokens were randomly masked, and we predicted the masked tokens. This procedure is known as masking the positions mechanisms. On that occurrence, the hidden vectors, which are ultimate and match the prescribed masked token, were fed into the SoftMax within the output layer above the reference points, employed as a standard position model.

### 3.6. Unscented Kalman Filter Tracking

The proposed system is defined through a non-linear model that depicts the association between the range of a target and the corresponding reference point or current position of a target. That is the main reason for applying a non-linear filtration algorithm, so we adopted the unscented Kalman filter (UKF). We used the unscented Kalman Filter over the extended and basic Kalman Filter because it is robust, fast, and performs much better than the latter [60,61]. The unscented Kalman Filter employs sigma points, which use points from the Gaussian source and then map them with the target’s Gaussian. The identified points are then passed through a non-linear function, calculating a new mean and transforming Gaussian variance. Since it is challenging to transform an entire state distribution using a non-linear function, it is easier to use individual points to transform the state distribution, which are sigma points. The sigma points represent the entire distribution. The unscented transformation uses the sample points, which manifest similar system state characteristics. The non-linear sample points applied by the unscented Kalman Filter are transformed using non-linear state and measurement equations.

Both the covariance and the mean are then calculated. Lastly, the combination of the estimated system states with the Kalman filter is recursively calculated. The sigma sample points are used as the distribution inputs. The UKF algorithm is used for tracking mobile targets, as illustrated in the expressions below, starting with prediction.

#### Prediction

*i.*  *The Initialization Stage*

(11) $$\left\{\begin{array}{l} {\hat{X}}_{0}=E\left\{X_{0}\right\}\\ P_{0}=E\left\{\left(X_{0}-{\hat{X}}_{0}\right){\left(X_{0}-{\hat{X}}_{0}\right)}^{T}\right\} \end{array}\right.$$

whereby $P_{0}$ is the representation of the preliminary covariance estimation error, and the initial state is represented by ${\hat{X}}_{0}$.

*ii.*  *The Generation of weights*

The dimensionality of the system determines the number of sigma points. The equation is expressed as follows 2N+1, which denotes the dimensions. With an assumption that the state of the variable is X of the N−dimensional vector, we can obtain the sigma-samples $\chi_{i}$ along with the related weight functions $W_{i}$.On the basis of covariance P and its variable X, we can update the state estimate and predict future states. The Generation of the Sigma sample points method is based on the usage of the unscented transform, as indicated below;

(12) $$\begin{array}{l} \chi_{k-1}^{\left(0\right)}={\hat{X}}_{t-1}\\ \chi_{t-1}^{\left(s\right)}={\hat{X}}_{t-1}+\sqrt{(n+\lambda )P_{t-1}},s=1,2,\cdots ,n\\ \chi_{t-1}^{\left(s\right)}={\hat{X}}_{t-1}-\sqrt{(n+\lambda )P_{t-1}^{\prime }},s=n+1,n+2,\cdots ,2n \end{array}$$

whereby χ is used to represent the Sigma Point Matrix. λ is used to denote the scaling factor, which is a deciding factor of the distance at which we should choose our sigma point in the meanwhile. The composite scaling factor is described as $\lambda =\alpha^{2}(n+t)-n$. The n is used to denote the dimensionality of the state. The tuning parameters are designated by the t and α.

*iii.*  *The state Prediction*

(13) $$\begin{array}{l} \chi_{t/t-1}^{\left(s\right)}=f\left(\chi_{t-1}^{\left(s\right)},k-1\right),s=0,1,2,\cdots ,2n\\ {\hat{X}}_{t/t-1}=\sum_{s=0}^{2n}\omega_{s}^{\left(m\right)}\chi_{t/t-1}^{\left(s\right)}\\ P_{XX}=\sum_{s=0}^{2n}\omega_{s}^{\left(c\right)}\left(\chi_{t/t-1}^{\left(s\right)}-{\hat{X}}_{t/t-1}\right){\left(\chi_{t/t-1}^{\left(s\right)}-{\hat{X}}_{t/t-1}\right)}^{L}\\ P_{t/t-1}=P_{XX}+\Gamma_{t-1}Q_{t-1}\Gamma_{t-1}^{L} \end{array}$$

whereby $\omega_{s}^{\left(q\right)}$ along with $\omega_{s}^{\left(r\right)}$ are described as follows;

(14) $$\begin{array}{l} \omega_{0}^{\left(q\right)}=\frac{\lambda }{n+\lambda }\\ \omega_{0}^{\left(r\right)}=\frac{\lambda }{n+\lambda }+\left(1-\alpha^{2}+\beta \right)\\ \omega_{s}^{\left(q\right)}=\omega_{s}^{\left(r\right)}=\frac{1}{2(n+\lambda )},s=1,2,\cdots ,2n \end{array}$$

whereby β is used as a way of integrating the information of the highest order with its distribution, with the Gaussian distribution being β=2 at its optimal.

*iv.*  *The Prediction Measurement*

(15) $$\begin{array}{l} \zeta_{t/t-1}^{\left(s\right)}=h\left(\chi_{t/t-1}^{\left(s\right)},t\right),s=0,1,2,\cdots ,2n\\ {\hat{Z}}_{t/t-1}=\sum_{s=0}^{2n}\omega_{s}^{\left(q\right)}\zeta_{t/t-1}^{\left(s\right)}. \end{array}$$

whereby ζ denotes

*v.*  *Calculating Kalman Gain*

(16) $$\begin{array}{l} P_{XZ.}=\sum_{s=0}^{2n}\omega_{s}^{\left(r\right)}\left(\chi_{t/t-1}^{\left(s\right)}-{\hat{X}}_{t/t-1}\right){\left(\zeta_{t/t-1}^{\left(s\right)}-{\hat{Z}}_{t/t-1}\right)}^{L}\\ P_{ZZ.}=\sum_{s=0}^{2n}\omega_{s}^{\left(r\right)}\left(\zeta_{t/t-1}^{\left(s\right)}-{\hat{Z}}_{t/t-1}\right){\left(\zeta_{t/t-1}^{\left(s\right)}-{\hat{Z}}_{t/t-1}\right)}^{L}+R_{t}\\ K_{t}=P_{XZ.}P_{ZZ.}^{-1} \end{array}$$

whereby

*vi.*  *Updating and Filtering*

(17) $$\begin{array}{l} {\hat{X}}_{t}={\hat{X}}_{t/t-1}+K_{t}\left(Z_{t}-{\hat{Z}}_{t/t-1}\right)\\ P_{t}=P_{t/t-1}-K_{t}P_{ZZ}K_{t}^{L}. \end{array}$$

For the next sampling, the algorithm executes the sigma points calculation step along with the update and filtering step.

## 4. Optimization Based on Position Algorithm

The proposed model utilized a classic optimization algorithm, the Stochastic Gradient Descent [62,63,64,65], with momentum performing much better than most used optimizers, such as Adams and the basic Stochastic Gradient Descent SGD [66]. The main reason for choosing this optimizer was that it works better and faster and aids in accelerating gradient vectors in the right direction, which speeds up converging. SGD and momentum utilize the exponentially weighted averages for dealing with sequences of the given numbers. Exponentially weighted averages are defined with the following expression when we have three successive new sequences of S derived from the original sequence O.

(18) $$\begin{array}{c} S_{t}=\beta S_{t-1}+(1-\beta )O_{t}\\ S_{t-1}F=\beta S_{t-2}+\left(1-\beta \right)O_{t-1}\\ S_{t-2}=\beta S_{t-3}+(1-\beta )O_{t-2} \end{array}$$

whereby t is the value at a particular position in the new sequence S, dependent on the original sequence O. The original sequence O is assigned weights. The weights are represented by beta β, which is multiplied by the t value of O. The method of getting the average of the sequences has been defined above. Next, we must apply the concept in our proposed model used as the moving average of our gradient, as indicated in the expression below.

(19) $$\begin{array}{c} s_{t}=\beta s_{t-1}+\alpha \nabla_{w}F(W,X,y)\\ W=W-s_{t} \end{array}$$

The loss function is represented by L, the nabla operator denotes the gradient weights, and the alpha indicates the learning rate.

## 5. Experimental Setup

### Experiments Description

**Figure 2:**
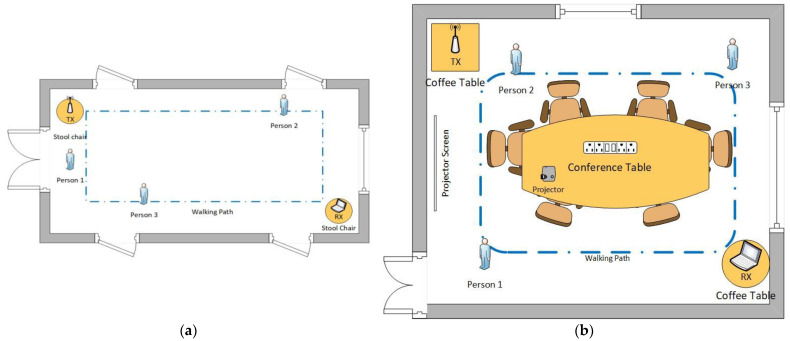
(**a**) The experimental testbed for collecting and processing raw CSI data. The experiment was conducted in a controlled environment at the University of Science and Technology, Beijing, using a laptop and Wi-Fi-router in the dormitory hallway. (**b**) The experiment was conducted in a controlled environment at the University of Science and Technology, Beijing, using a laptop and Wi-Fi-router in the conference room.

Figure 2a,b below shows the data collection layout for the proposed system experiments, which were performed using a gigabyte laptop equipped with a Wi-Fi network interface card by intel model Link 5300 802.11n, which was used as a receiver (RX). The computer used modified wireless network drivers to collect the CSI data through the open-source Linux Ubuntu 18.04 LTS operating system. We used a TP-Link TL-WR886N wireless access point which acted as a signal transmitter (TX). A moving target being tracked has to be in the line of sight, as indicated by Figure 2a,b, representing the testbeds for experiments.

The CSI data we collected while tracking the 20 participants was 17,600 = (10 min × 60 s/min × 20 Hz), with 880 for each participant. The collected samples were acquired from a controlled environment set up as shown in Figure 2a,b below, using only two devices: the laptop as an RX and the access point as TX. Participants were randomly asked to walk around the room, not following a specific path. While walking, a person has a unique reflection and influence on the radio signal. Several studies have indicated a reduced obstruction when the radio signal propagates freely in open spaces. Hence, we placed the RX and TX 1 m above the ground level to overcome this obstruction drawback. We collected the sample data in the dormitory four-floor 12 hallway and the School of Computing conference room at the University of Science Technology, Beijing. The participants walked different distances in meters from the entrance through the predefined five routes, both in the hallway and conference room.

The experimental testbeds used were divided into a grid system. Each grid is a representation of an average step size of 0.8 m, which are marked as positions. For example, the hallway measurements were a length of 8 m, which made up 10 positions, and a width of 5 m, which made up 5 positions, meaning the room had an overall of 50 positions. On the other hand, the conference room measurements were a length of 8 m, which made up 10 positions, and a width of 6 m, which made up 7.5 positions, meaning the room had an overall of 75 positions. In addition, some of the grids were covered by the conference table, which was 4 by 2 and covered a grid of 15.625, leaving a space of 59.4 positions.

For the collection of the data set, we used 20 participants to walk on the predefined routes for both test beds since they had matching settings. The predefined routes consisted of various patterns, including walking in circles, walking in a straight line, walking in a zigzag manner (going forward, backwards, and sideways), walking in a spiral pattern (gradually moving closer or farther away from a fixed point), and walking in a rectangular pattern.

All the activities on both testbeds, including participants walking on each predefined route, were recorded on a camera for four days. The main reason for recording these activities was to ensure that the participants were honest and to gather additional information about their movements. This allowed us to observe if participants were correctly following the predefined routes. This comprehensive approach allowed us to collect accurate and reliable CSI data for further analysis and modeling.

Machine learning models usually use vast data to perform better with higher accuracy. The collected sample data for the project is augmented to increase the effectiveness of the dataset. For instance, walking is a continuous activity. Therefore, we split data with long sequences into shorter lengths. Since walking is a symmetric activity, we reversed some features within the augmented time domain. Training the proposed model was conducted with 70% of the collected sample data, and the remaining 30% was used to test the accuracy of the models. The proposed model was tested using a desktop computer equipped with an Intel (R) Xeon(R) CPU E5-2660 0 @ 2.20GHz 2.20 GHz processer with 16.0 GB DDR 3 ram, and an Nvidia GeForce GTX 1060 GPU card. The results obtained from training and testing the proposed model are presented in Section 4.

## 6. Simulations and Results

**Figure 3:**
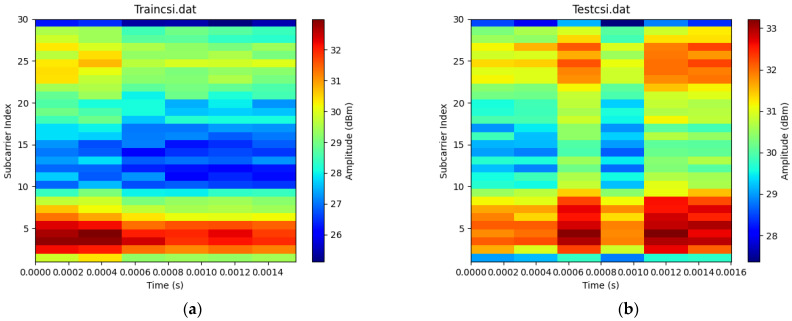
(**a**) Colormap for raw CSI data of a target moving forward in a conference room taken in 0.0014 microseconds. (**b**) Colormap for raw CSI data of a target moving forward in a dormitory hallway room taken in 0.0016 microseconds.

The validation of the proposed model utilizes the collected CSI data to test the system’s performance while tracking both single and multiple targets [61]. Figure 2 and Figure 3 indicate the simulation data collection setups. The collected CSI data was calibrated for noise reduction. Figure 3a,b below shows a colormap diagram of raw CSI data before calibration. The data was collected in microseconds to capture small movements by the target and use them for measuring instantaneous accelerations.

**Figure 4:**
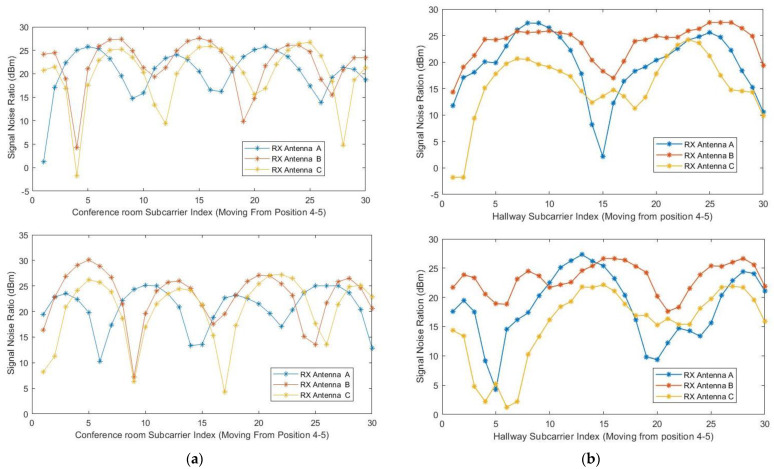
(**a**) Channel state information was measured in line–of–sight condition for three antennas as a function of the subcarrier index for a target moving in a straight line and backwards from position 4 to 5 on the conference room-controlled testbed. (**b**) Channel state information was measured in line–of–sight condition for three antennas as a function of the subcarrier index for a target moving in a zigzag and sideways from position 2 to 3 on the hallway-controlled testbed.

Figure 4a,b below serve as illustrations of the variations in signal amplitude resulting from the target’s movement in the testbeds. These figures visually demonstrate the unique amplitude patterns observed at different positions. The analysis of these figures underscores the importance of leveraging CSI signals, as indicated by the conclusion drawn regarding the favorable results achieved through matching specific CSI parameters. These figures visually demonstrate the changes in signal amplitude as the target moves within the conference room and dormitory hallway testbed. Each position within the testbeds exhibits a distinct amplitude pattern that corresponds to the target’s distance from the transmitter and receiver. The focus here is on the utilization of CSI signals to optimize signal processing techniques and enhance overall system performance. Channel state information comprises time stamps, which enfold every 4300 s (or 72 min), the beamforming count registered by the drives, and the transmitter-receiver information. Additionally, the Channel State Information (CSI) includes not only RSSI but also permuted signals from the three receive antennas, packet rates, and the 3D matrix of the OFDM subcarriers. These elements, as illustrated in the figures provided, form the comprehensive CSI dataset, enabling more accurate analysis of the wireless channel’s characteristics and behavior.

**Figure 5:**
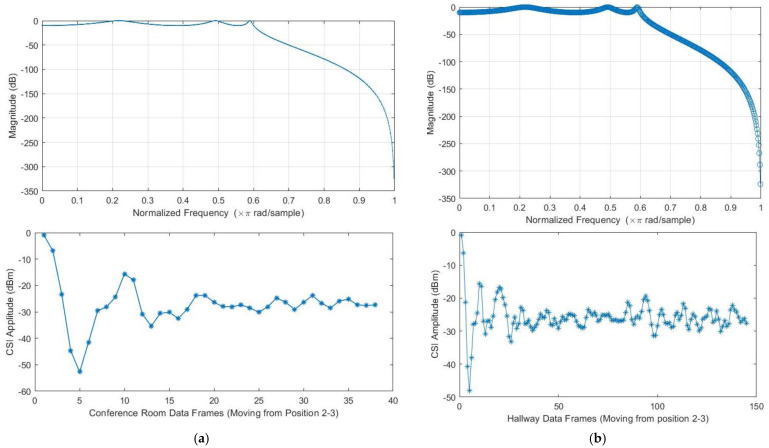
(**a**) Target moving in a straight line from position 2 to position 3 on the conference room test bed with a filter is detected by the phase differences across adjacent antennas. (**b**) Detection of a target moving in a straight line by the phase differences across adjacent antennas from position 2 to position 3 on the hallway test bed.

**Figure 6:**
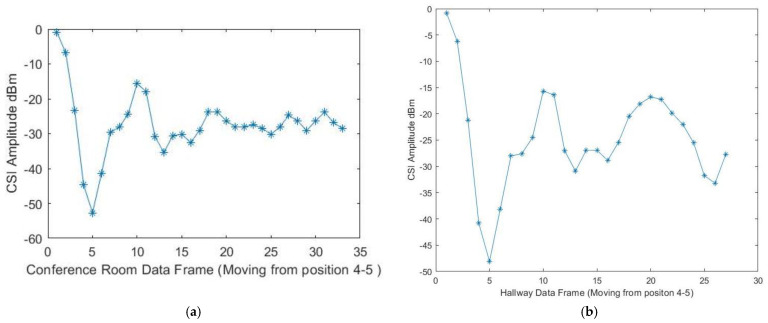
(**a**) Detection of a target moving in a straight line by the phase differences across adjacent antennas from position 4 to position 5 on the conference room test bed. (**b**) Detection of a target moving in a rectangular pattern by the phase differences across adjacent antennas from position 4 to position 5 on the hallway test bed.

Figure 5a,b below demonstrate the target motion detection by the phase differences across adjacent antennas through a filter from one position to another in the conference room testbed. The Wi-Fi Doppler effect may be ambiguous because the target changes the path length while walking parallel to the line of sight of the propagating signal from the transmitter to the receiver antennas. We asked the participants to consecutively walk in the same direction at a constant speed to determine the Wi-Fi Doppler effect based on their natural walking pattern. This allowed us to obtain the target’s estimated directional speed optimally. When there is no movement within the line of sight, there will be no Doppler effect, so the phase difference will be stationary. Nevertheless, as indicated in Figure 6a,b, the phase differences fluctuate enormously when the target is in motion. We put up a threshold to denote motion. Therefore, when phase differences are less than the threshold, we would know that movement is impossible. Contrary to that, we would know that the target is moving. To validate the proposed system’s ability to accommodate various participants, we used ten volunteers as our subjects. We had four females and six males between the ages of 19 and 30 years old and with heights of 150 to 188 cm to capture our datasets, which were taken on four different days (two days on each testbed). The training data that we collected was tested four times. The CSI data that we collected for all the participants was divided, and we used 70% of the collected dataset for training, and the remaining 30% was utilized for testing. The performance evaluation metrics of the proposed included the error matrix, which allowed us to measure the proposed systems to measure recall, precision, accuracy, and area under the curve–receiver operating characteristics (AUC–ROC) curve. We describe performance evaluation metrics in detail in the subsection below.

### 6.1. Performance Evaluation of Proposed and Existing Methods

The performance evaluation metric adopted for our research study includes the confusion matrix focused on the true negative rate (TN), which is the probability that a nonmoving target is classified correctly. In our research study, we evaluated the overall accuracy of our proposed machine learning model using confusion matrices and focused on specific performance metrics such as the true positive (TP) rate and F1 score. The TP rate measures the probability that a moving target is correctly detected and classified as positive by the model. This metric is crucial in object detection and tracking applications, where correctly identifying moving targets is essential. In our study, we calculated the TP rate by dividing the number of true positives by the total number of positive instances. We collected datasets from two different environments, namely the hallway and conference room, and evaluated the performance of our model in each environment.

**Figure 7:**
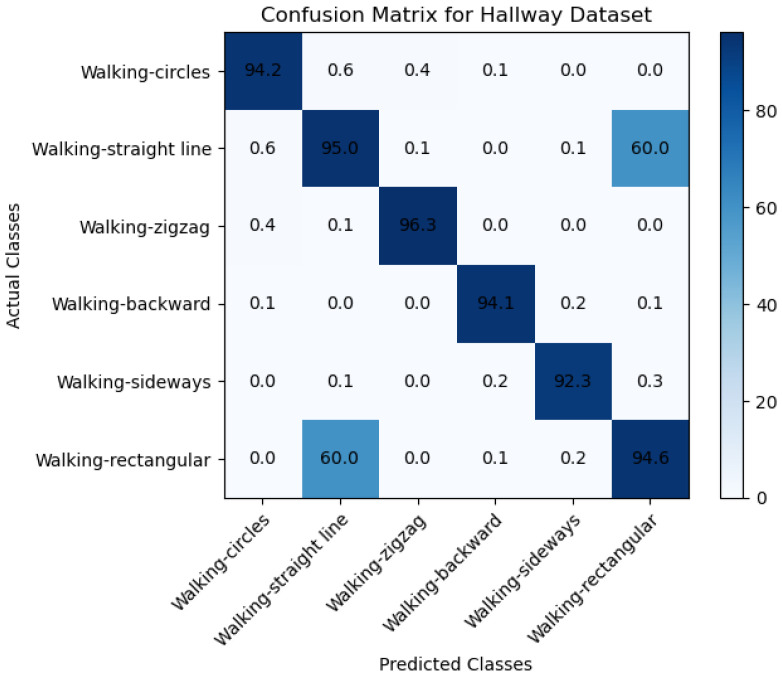
Confusion Matrix to evaluate the performance of the proposed model with the collected hallway dataset.

**Figure 8:**
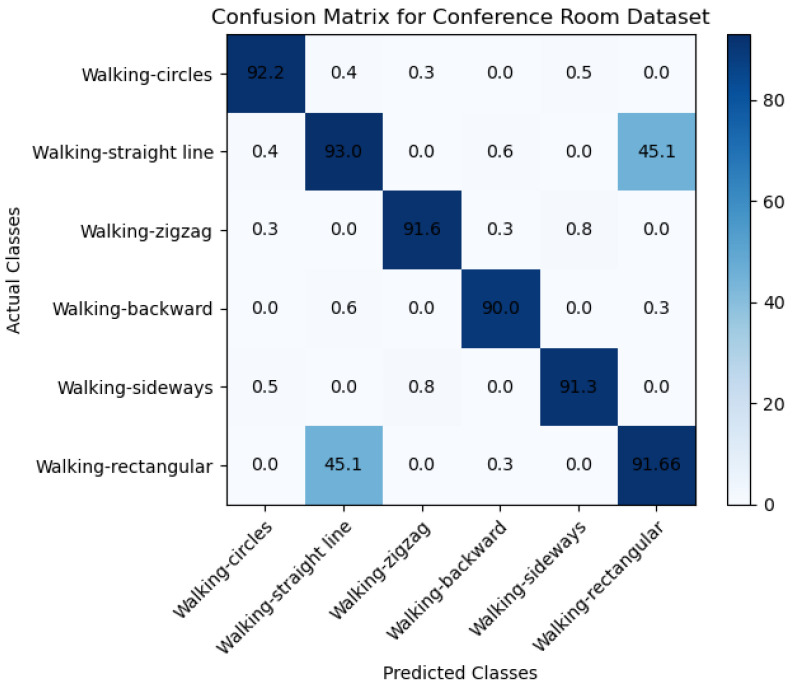
Confusion Matrix to evaluate the performance of the proposed model with the collected conference room dataset.

To analyze the results, we created confusion matrices for each environment presented in Figure 7 and Figure 8. These matrices provided a detailed breakdown of the model’s performance regarding true positive and false positive rates. In addition to the TP rate, we adopted the F1 score as another performance metric in our study. The F1 score is a weighted average of the precision and recall, providing a balanced measure of the model’s accuracy. The F1 score ranges from 0 to 1, where a score of 1 represents perfect accuracy. To calculate the F1 score, we used precision and recall values from the confusion matrices. Precision measures the proportion of true positive predictions among all positive predictions, while recall measures the proportion of true positive predictions among all actual positive instances. The F1 score is then calculated using the formula 2 × (precision × recall)/(precision + recall).

Moreover, the TP rate and F1 score allowed us to evaluate the model’s performance more nuanced and detailedly, beyond the overall accuracy measured through the confusion matrices. Specifically, the TP rate was a key metric in evaluating the model’s effectiveness in correctly detecting moving targets. At the same time, the F1 score provided a balanced measure of the model’s accuracy that considered both precision and recall. By analyzing these multiple metrics, we gained a more comprehensive understanding of the model’s strengths and weaknesses, which can help us make informed decisions on further improving and optimizing the model for real-world applications.

Table 1 and Table 2 below show the evaluation metrics results for the self-collected data sets in different environments. For the conference room dataset in Table 1, the results show that the recall that was archived was 0.90, which means that the model correctly identified 90% as positive, and 10% were incorrectly classified as negative (false negatives). This means that the model effectively detects positive cases when they are present in the dataset. Table 1 also includes an F1 score of 0.91, meaning the model has achieved a balance between precision and recall. The F1 score is a harmonic mean of precision and recall, combining both metrics into a single score. This means that the proposed model with an F1 score of 0.91 (which is a good score) is performing well on the dataset.

Moreover, the other dataset collected from the hallway environment, as indicated in Table 2, archived a recall of 0.95, an F1 score of 0.95, a precision of 0.96, and a weighted value of 0.946. This means that the model has achieved high performance in terms of precision and recall, with both metrics above 0.95. The F1 score, a weighted harmonic mean of precision and recall, is also high at 0.96. Moreover, the increased weighted average of 0.946 suggests that the model performs well across all classes, even when considering differences in sample sizes between classes. This is important in cases where the dataset is imbalanced and some classes have more samples than others.

### 6.2. Comparative Analysis of the Proposed Model with Other Models

In this section, we present a comparative analysis of several machine learning models for predicting the position and tracking of a moving target in two different environments, as shown in Table 3 and Table 4. We evaluate the performance of Long Short-Term Memory Networks (LSTM), Support Vector Machine (SVM), k-nearest neighbors’ algorithm, and recurrent neural networks (RNNs), as well as a proposed model based on self-attention mechanisms. We compare the model’s accuracy levels with 100 epochs during the training and testing phases. Our main goal is to identify each model’s strengths and limitations and provide insights into the potential of deep learning models for predicting the position and tracking of a moving target. Based on the conference room dataset results, the proposed model outperformed the other models in both training and testing accuracy. The proposed model achieved 97% training accuracy and 96% testing accuracy, indicating that it could learn the patterns in the data well and generalize to new data.

Among the other models, as indicated in Table 3 for the hall environment dataset, the RNN performed the second best, achieving 91.9% training accuracy and 90.3% testing accuracy. The KNN model also performed relatively well, achieving 90.2% training accuracy and 88.9% testing accuracy. The LSTM model achieved 90.1% training accuracy and 89% testing accuracy, while the SVM model achieved 81.1% training accuracy and 80% testing accuracy.

Therefore, it can be concluded that the proposed model is the most effective model for this dataset, followed by the RNN and KNN models. The LSTM and SVM models performed relatively poorly compared to the other models. It is important to note that other factors, such as computational efficiency, interpretability, and model complexity, were also considered when choosing a model for this task.

We further tested the same models for the same task using some datasets collected in a different environment, as indicated in Table 4 above. With 93.00% training accuracy and 91.66% testing accuracy, the proposed model outperformed all other models in terms of training and test accuracy. This suggests that the proposed model can effectively generalize to new data and learn patterns from the hallway dataset.

## 7. Results Discussion

The main aim of this research study was to use a device-free target tracking method and an unscented Kalman filter with Wi-Fi signals in the form of channel state information and incorporate the self-attention mechanism model. The preceding sections provide a detailed theoretical exposition of the key contributions of the research, which are informed by the results presented in subsections A, B, and C. We intended to develop a novel fine-grained target-tracking model that utilizes self-attention and CSI to passively locate and track moving targets while estimating their position with instantaneous acceleration. The model employs batch processing and Kalman filtering for online tracking, improving position estimation accuracy over time. Additionally, we propose using an unscented Kalman filter for instant position estimation of multiple targets. Our experiments, conducted in real-time using commodity Wi-Fi devices on a custom-designed platform, validate the performance of our proposed model. Figure 4a,b and Figure 5a,b depict the movement of various targets from one position to another in a raw CSI waveform. These Figures illustrate the changes in amplitude and phase of the received Wi-Fi signals as the targets move and how these changes are used to locate and track the targets. Specifically, each Figure shows the movement of one target over time, with different sub-Figures representing different time points. The raw CSI waveform is plotted on the *y*-axis, while the *x*-axis represents time. On the other hand, Figure 3a,b are presented in a colormap format, which allows for a more visual representation of the movement of the targets. The color map represents the amplitude and phase changes of the Wi-Fi signals in different colors, with each color representing a different value. These Figures show the movement of multiple targets simultaneously, with different colors indicating different targets. These Figures provide valuable insights into the proposed fine-grained target-tracking approach, demonstrating its ability to locate and track multiple targets using Wi-Fi signals accurately. Figure 5a,b illustrate the movement of targets after applying the unscented Kalman filter to the raw CSI data. The unscented Kalman filter is a data processing technique that helps estimate the state of a system based on noisy sensor measurements. In the context of target tracking, the unscented Kalman filter can help improve position estimation accuracy by reducing the impact of noise and other sources of uncertainty in the CSI data. The filtered CSI data was then used to build datasets for moving targets in two different environments. These datasets contain information about the targets’ movements, including their position, velocity, and acceleration, over time. The two environments in question were custom-designed indoor spaces that were specifically designed to test the performance of the proposed tracking approach. Overall, Figure 5 and Figure 6 provide valuable insights into the effectiveness of the unscented Kalman filter in improving target tracking accuracy. By comparing the filtered CSI data to the raw CSI data, we can see how the filter helps reduce noise and improve the overall quality of the data. Additionally, the datasets generated from the filtered CSI data provide a valuable resource for further research and experimentation in target tracking using Wi-Fi signals. To evaluate the effectiveness of the proposed system, we used various evaluation metrics derived from the confusion matrix. These metrics include accuracy, precision, recall, specificity, F1 score, and AUC–ROC curve. The evaluation results are presented in Figure 7 and Figure 8 and Table 1, Table 2, Table 3 and Table 4. So, the overall evaluation of the results suggests that the proposed model is highly effective in identifying and tracking moving targets using commodity Wi-Fi devices. The high accuracy level achieved by the model indicates that it can reliably detect and track most of the targets. In contrast, the high precision and recall scores suggest that the model can accurately identify true positives and negatives. These findings are significant, as they demonstrate the potential for using Wi-Fi signals for target tracking in a wide range of applications, including surveillance, security, and human-computer interaction.

## 8. Conclusions and Future Work

This study’s results demonstrate the proposed model’s effectiveness for target tracking using commodity Wi-Fi devices. The high accuracy level achieved by the model indicates that it can reliably detect and track most targets. The high precision and recall scores demonstrate that the model accurately identifies true positives and negatives. These findings have important implications for a wide range of applications, including surveillance, security, and human-computer interaction.

In conclusion, this study has proposed a novel target-tracking approach using Wi-Fi signals and a sole self-attention mechanism. The proposed model utilizes batch processing and particle filtering techniques to track the targets and estimate their positions accurately. Additionally, the model uses an unscented Kalman filter to estimate the instant positions of multiple moving targets. The results of this study indicate that this approach is highly effective for target tracking, achieving an accuracy level of 97%.

In terms of future work, further research could be conducted to explore the potential of this approach for real-time target tracking in complex and dynamic environments. Additionally, the proposed model could be extended to incorporate other sources of data, such as visual or acoustic signals, to enhance the accuracy and robustness of the tracking system. Finally, the proposed model could be optimized to improve its computational efficiency, making it suitable for deployment in resource-constrained environments.

## Figures and Tables

**Table 1:** Evaluation Matrices for the Proposed Model (Conference Room Dataset).

Metrics	Measures
Recall	0.90
F1 Score	0.90
Precision	0.91
Weighted Average	0.9166

**Table 2:** Evaluation Matrices for the Proposed Model (Hallway Dataset).

Metrics	Measures
Recall	0.95
F1 Score	0.95
Precision	0.96
Weighted Average	0.946

**Table 3:** Results for dormitory hallway data set.

Models	Epochs	Training Accuracy	Testing Accuracy
Proposed Model	100	97.0	96.0
LSTM	100	90.1	89.00
SVM	100	81.1	80.0
KNN	100	90.2	88.9
RNN	100	91.9	90.3

**Table 4:** Results for conference room data set.

Models	Epochs	Training Accuracy	Testing Accuracy
Proposed Model	100	93.00	91.66
LSTM	100	89.9	88.00
SVM	100	82.10	80.00
KNN	100	91.00	90.00
RNN	100	92.00	90.66

## Data Availability

Not applicable.
